# Methylation‐reprogrammed Wnt/β‐catenin signalling mediated prenatal hypoxia‐induced brain injury in foetal and offspring rats

**DOI:** 10.1111/jcmm.13660

**Published:** 2018-05-28

**Authors:** Yingying Zhang, Mengshu Zhang, Lingjun Li, Bin Wei, Axin He, Likui Lu, Xiang Li, Lubo Zhang, Zhice Xu, Miao Sun

**Affiliations:** ^1^ Institute for Fetology First Hospital of Soochow University Suzhou China; ^2^ Center for Perinatal Biology Loma Linda University Loma Linda CA USA

**Keywords:** brain, methylation, prenatal hypoxia, Sfrp4, Wnt

## Abstract

Prenatal hypoxia (PH) is a common pregnancy complication, harmful to brain development. This study investigated whether and how PH affected Wnt pathway in the brain. Pregnant rats were exposed to hypoxia (10.5% O_2_) or normoxia (21% O_2_; Control). Foetal brain weight and body weight were decreased in the PH group, the ratio of brain weight to body weight was increased significantly. Prenatal hypoxia increased mRNA expression of Wnt3a, Wnt7a, Wnt7b and Fzd4, but not Lrp6. Activated β‐catenin protein and Fosl1 expression were also significantly up‐regulated. Increased Hif1a expression was found in the PH group associated with the higher Wnt signalling. Among 5 members of the Sfrp family, Sfrp4 was down‐regulated. In the methylation‐regulating genes, higher mRNA expressions of Dnmt1 and Dnmt3b were found in the PH group. Sodium bisulphite and sequencing revealed hyper‐methylation in the promoter region of Sfrp4 gene in the foetal brain, accounting for its decreased expression and contributing to the activation of the Wnt‐Catenin signalling. The study of PC12 cells treated with 5‐aza further approved that decreased methylation could result in the higher Sfrp4 expression. In the offspring hippocampus, protein levels of Hif1a and mRNA expression of Sfrp4 were unchanged, whereas Wnt signal pathway was inhibited. The data demonstrated that PH activated the Wnt pathway in the foetal brain, related to the hyper‐methylation of Sfrp4 as well as Hif1a signalling. Activated Wnt signalling might play acute protective roles to the foetal brain in response to hypoxia, also would result in disadvantageous influence on the offspring in long‐term.

## 1 INTRODUCTION

The hypothesis on foetal origins of adult diseases (FOAD) also known as the developmental origins of health and disease (DOHaD) states that the later‐onset illness such as cardiovascular diseases, diabetes type II, neurologic disorders and cancers are associated with adverse environmental exposure such as hypoxia in foetal and infant life.^1^, ^2^ It is widely accepted that the brain is extremely sensitive to hypoxia. Exposed to hypoxia can result in cognitive dysfunction, spatial learning and memory impairment and anxiety‐like behaviour.^3^, ^4^ Prenatal hypoxia is one of the most common pregnancy complication and the main cause of foetal death. Perinatal asphyxia induces the activation of multiple cell death mediators such as death receptors, Bcl‐2 and caspase protein families, with increased apoptosis in the brain. Following perinatal asphyxia, microglia is activated and induces local inflammation in the brain and over‐activation of microglia can aggravate neuronal cell death.^5^ Perinatal hypoxia‐caused ischaemia can induce damage of central grey matter, which is linked to death during the neonatal period (20%‐30%). Among survivors, children showed an increased risk of feeding difficulty, speech and communication problems, visual impairment, hearing loss, epilepsy and cerebral palsy.^5^, ^6^ Furthermore, prenatal or perinatal hypoxia is regarded to be involved in the development of attention‐deficit hyperactivity disorder (ADHD) and is correlated with autism in childhood.^7^, ^8^ Children exposed to perinatal asphyxia demonstrated a higher risk of developing ADHD than unexposed children.^9^ Recent work showed prematurity, post‐term birth, pre‐eclampsia and prenatal or perinatal hypoxia were correlated with autism.^10^ Furthermore, the nucleus tractus solitarius (NTS) is highly sensitive to hypoxia. In autism, the NTS is impaired because of potentiated micro‐circulatory insufficiency.^11^ Our previous studies have demonstrated that Prenatal hypoxia (PH) damaged spatial acquisition abilities in the adolescent rat offspring via down‐regulating of Wnt/β‐catenin signalling in the hippocampus, however, the underlying mechanism is not clear yet.

Wnt/β‐catenin signalling plays an important role in the brain, including axonal remodelling and patterning, neurogenesis and development of functional synapses within the CNS.^12^, ^13^, ^14^ Abnormal Wnt/β‐catenin signalling which is usually down‐regulation has been shown to participate in the onset/development of neurodegenerative disorders, including Alzheimer's Disease (AD) and Parkinson's disease (PD). In AD, down‐regulated Wnt/β‐catenin signalling induced abnormal processing of amyloid precursor protein (APP) (the main cause of AD), whereas in PD, its down‐regulation leads to the progressive loss of midbrain dopaminergic (mDA) neurons.^15^, ^16^ Activated Wnt/β‐catenin has been found mainly in cancers such as glioblastoma, ovarian cancer, cervical cancer, medulloblastoma and hepatocellular carcinoma.^17^, ^18^, ^19^, ^20^, ^21^ In hepatocellular carcinoma, Wnt signalling was activated by hypoxia, a common feature of solid tumours, through regulating the expression of BCL9 (an essential co‐activator in the Wnt signalling). Furthermore, evidence have suggested that hypoxia induced activation of the Wnt signalling and stimulated cell proliferation and neurogenesis in the mice hippocampus.^22^ If Hif1a, a protein induced by hypoxia, was knocked down under hypoxic conditions, the cobalt‐mimicked hypoxia could not induce the activation of the Wnt signalling in MC3T3‐E1 cells (the mice osteoblast‐like cell line). This result confirmed that the activation of the Wnt signalling is dependent on Hif1a.^23^ Our laboratory reported that Wnt/β‐catenin was decreased in the brain of the adolescence offspring exposed to prenatal hypoxia, with long‐term effects on hippocampal neurogenesis and functions. However, why prenatal hypoxia could result in decreased Wnt/β‐catenin signals needs further investigation.^24^

Recently, epigenetics has developed rapidly and is related with foetal origins of adult diseases. Epigenetic mechanisms regulate many aspects of cellular function such as transcription and post‐translation modification.^25^ Intrauterine environmental exposures (hypoxia, nicotine, malnutrition) could result in epigenetic code in prenatal period inducing dysregulated gene expression, which leading to the developmental origins of several human chronic diseases including hypertension, coronary heart disease, stroke, diabetes, neural and mental disorders and cancer.^26^ This study investigated Wnt signals in both foetal and offspring brain, and proposed that the foetal brain responds to PH by activating Wnt/β‐catenin signals which involved in methylation regulation. Abnormal Wnt/β‐catenin signal epigenetic reprogramming could be a potential cause of FOAD in neural systems.

## 2 MATERIALS AND METHODS

### 2.1 Animals

Sprague‐Dawley rats (3 months old) were obtained from Soochow University Experimental Animal Center and housed in a controlled environment at 22°C with a 12/12 hour light‐dark cycle. Food and water were available ad libitum. One female rat was mated with 2 male rats. When vaginal plugs were observed in the following morning, the day was recorded as the first day of gestation (GD 1). Pregnant rats were randomly divided into 2 groups: The control group was kept in normal atmospheric conditions (21% O_2_; normoxia) throughout pregnancy. The other group was exposed to hypoxia (10.5% O_2_; hypoxia) in a plexiglass chamber from GD 4 to 21. At GD 21, some of pregnant dams were anaesthetized intraperitoneally with sodium pentobarbital (100 mg/kg, intraperitoneally), foetuses were removed through surgical hysterotomy and weighed immediately. The foetal brain was isolated and weighed, then frozen in liquid nitrogen and stored in −80°C freezer. Other pregnant rats were allowed to give birth naturally. The male offspring at 6‐week old were used for testing. All experimental procedures and protocols were approved by the Institutional Animal Care and Use Committee and in accordance with the Guidelines for the Care and Use of Laboratory Animals.

### 2.2 Western blot analysis

Foetal forebrain samples, offspring hippocampus samples and PC12 cells were disrupted in 500 μL RIPA buffer added with PMSF, and then were held on ice for 30 minutes. After centrifugation at 13 800 g for 30 minutes at 4°C, the clean extracts were obtained and subjected to Western blot analysis. Protein (50 μg) were loaded to 10% SDS‐PAGE gels and transferred to PVDF membrane. Hif1a, β‐catenin, active‐β‐catenin and actin protein abundance were measured using primary antibodies (Hif1a, 1:1000, Thermo Scientific), (β‐catenin, 1:2000, Millipore), (active‐β‐catenin, 1:2000, Millipore) and (actin, 1:2000, Beyotime); Blots were visualized using chemiluminescence detection (Amersham Biosciences, Piscatawy, NJ, USA). Imaging signals were digitized and analysed with UVP imaging system (Tanon‐5200, Shanghai, China), and relative density of bands was normalized to actin as a control.

### 2.3 Real‐time quantitative PCR

Total RNA was extracted from foetal forebrain and offspring hippocampus using RNAiso Plus (TaKaRa). Purified total RNA (500 ng) was then reversely transcribed using the RevertAid First Strand cDNA Synthesis Kit (Thermo Scientific) following the manufacturer's instructions. Primers for q‐PCR assays were designed using the Primer Express 4.0 software (Table. S1). q‐PCR was performed with gene‐specific primers, cDNA and SYBR Premix Ex Taq (TaKaRa) using a Bio‐Rad icycler iQ. Each assay was repeated 3 times and the relative levels of mRNA were normalized to the control actin using the 2 ^−ΔΔCT^ method.

### 2.4 Methylation analysis

Genomic DNA from brain tissue samples was extracted using phenol:chloroform:isoamyl alcohol (25:24:1, Solarbio) and treated with sodium bisulphite with EZ DNA Methylation™‐GOLD Kit (Zymo Research) according to manufacturer's protocols. Unmethylated cytosine residues were converted to thymines, whereas methyl‐cytosines remain unmodified. PCR amplification was prepared for target CpG regions. After PCR amplification and library construction, products were sequenced on the Illumina MiSeq platform according to manufacturer's protocols. Methylation levels of 32 CpG sites in Sfrp4 were measured. Each tested CpG site was named according to its relative distance (in bp) to transcriptional start site (TSS). The percentage of methylation level of each CpG site was calculated as the ratio of methylated cytosines/total tested cytosines. The average methylation level was calculated using methylation levels of all measured CpG sites within the gene.

### 2.5 Morris water task

Spatial learning and memory in offspring was assessed using Morris water amazes. It consisted of 2 parts: a submerged platform (26 cm in height and 10 cm in diameter) and a circular pool (0.46 m in depth and 1.2 m in diameter). The rats were trained for 7 days with 4 acquisition trials each day. In each trial, rats were placed in the pool facing towards the wall. It was ended until it found the hidden platform, or until a limited time (60 seconds for a rat) elapsed, then the rat was guided to the platform and stay on it for 15 seconds. Swim speed, latency and travel distance to reach the platform were recorded. At the end of the day of acquisition training, the probe test to evaluate reference memory component in rats was used after the hidden platform was removed. The number of target approaches was recorded. All activities of rats in the tests were monitored, recorded and analysed using MT‐200 water maze video tracking system (Taimeng, Chengdu, China)

### 2.6 Cell culture and 5‐aza‐2′‐deoxycytidine treatment

PC12 cells (the rat pheochromocytoma cell line, extensively used model to study neuronal differentiation) were cultured at 37°C in Dulbecco's modified Eagle's medium (DMEM, High Glucose supplement) containing 10% (v/v) heat‐inactivated foetal bovine serum (HyClone, Utah State, USA) and 100 mg/mL penicillin G and 100 mg/mL streptomycin sulphate. After seeded in 6 cm dishes overnight, cells were treated with 5‐aza‐2′‐deoxycytidine (5*10^−7 ^mol/L) for 48 hour before harvesting. Expression of Dnmts, Sfrp4, Wnt3a, Wnt5a, Fzd4, Lrp6 and Catenin were examined by q‐PCR or Western Blot.

### 2.7 Data analysis

The program Prism (GraphPad) were used to analyse the data. The 2‐way analysis of variance (ANOVA) was conducted to analyse the water maze data. Other data were determined by the Student's *t*‐test. *P* < .05 was considered statistically significant. The results were expressed as mean ± SEM.

## 3 RESULTS

### 3.1 Impact of PH on foetal brain and body weight

Foetal brain and body weight were measured at GD 21. Prenatal hypoxia reduced brain and body weight, indicating growth delay and poor brain development (Figure S1A, B). The ratio of brain weight to body weight in the PH was increased (*P* < .05) (Figure S1C). Blood oxygen level and oxyhemoglobin saturation were significantly decreased in foetal blood in the PH. (*P* < .05) (Table. S2).

### 3.2 Hif1a and Wnt/β‐catenin pathway was up‐regulated in the foetal brain exposed to PH

**Figure 1:**
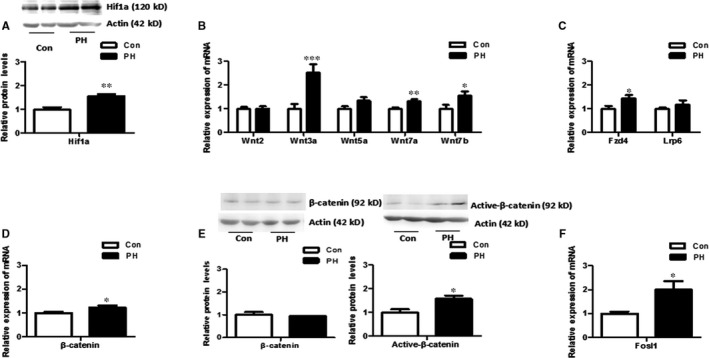
Hif1a and Wnt signalling levels in the foetal brain exposed to PH. A, Hif1a protein level was increased in the PH; n = 8 from 4 litters. B, C, Wnt3a, Wnt7a, Wnt7b and Fzd4 expression levels were increased in the PH group. D, β‐catenin mRNA expression was increased in the PH; n = 18 from 9 litters. E, Active form of β‐catenin protein was increased in the PH, whereas total β‐catenin protein showed no difference. n = 8 from 4 litters. F, The expression of Fosl1 was significantly increased in the PH. n = 10 from 5 litters; *P < .05; **P < .01; Con: control; PH: prenatal hypoxia

Hif1a protein was increased in the foetal brain of PH group (*P* < .01) (Figure 1A). The q‐PCR results showed that the expression of Wnt3a, wnt7a and wnt7b in foetal brain was increased in the PH group (*P* < .01), whereas there was no difference in Wnt2 and Wnt5a (Figure 1B). In the PH group, the expression of Frizzled protein 4 (Fzd4) was increased (*P* < .05), whereas Lrp6 was unchanged (Figure 1C). Both expression of mRNA and active form of β‐catenin protein were increased significantly (*P* < .05) in the PH group, whereas total β‐catenin protein remained unchanged (Figure 1D, E). Fosl1, a directly down‐stream gene of Wnt signalling, was increased in the foetal brain in the PH group (*P* < .05) (Figure 1F). These results showed that Wnt signalling was activated in the foetal brain exposed to PH.

### 3.3 Sfrp4 was hypermethylated and down‐regulated in the foetal forebrain exposed to PH

**Figure 2:**
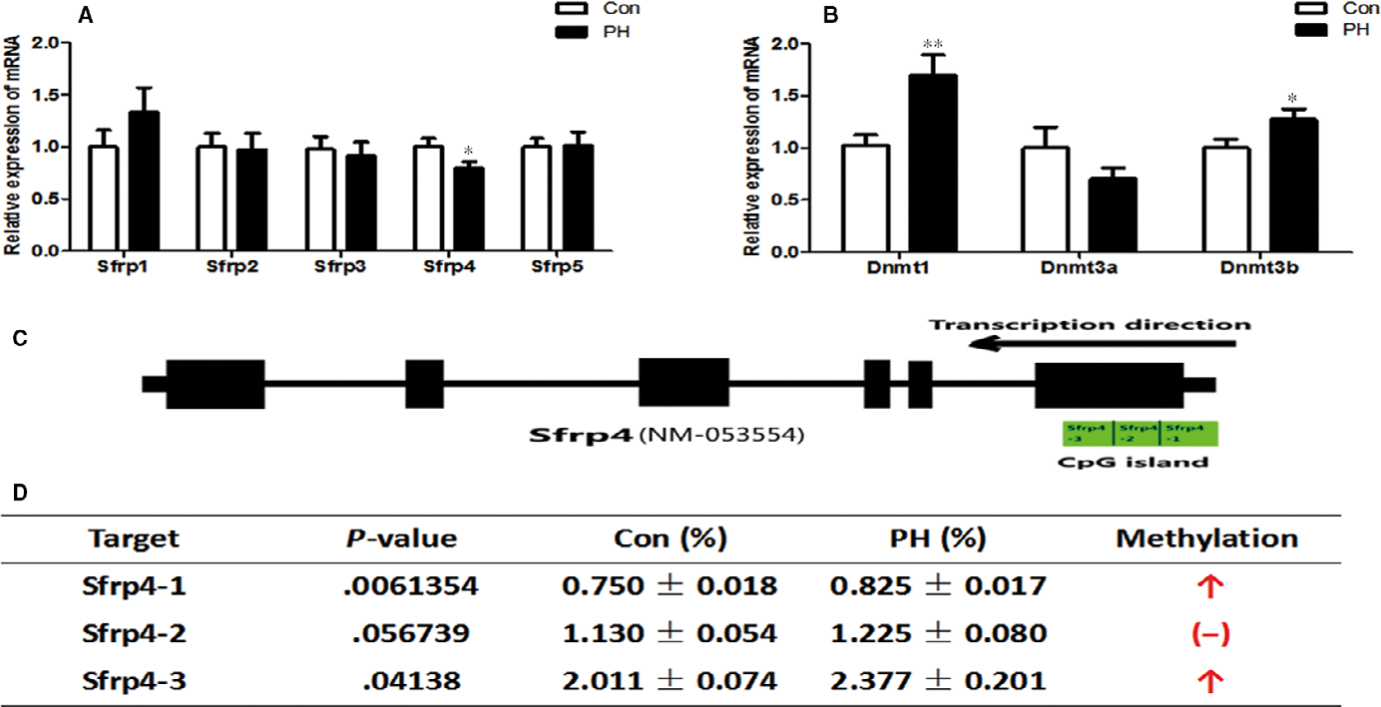
Expression and methylation levels of Sfrp4 in the foetal brain exposed to PH. A, Sfrp4 mRNA expression was decreased in the PH, whereas other Sfrps were not changed. n = 18 from 9 litters; B, Dnmt1 and Dnmt3b expression levels were increased in the PH. n = 18 from 9 litters; C, Three regions from CpG islands of Sfrp4 were sequenced. D, The methylation level of Sfrp4 was increased in the PH compared with the Con. n = 5 from 3 litters; *P < .05; **P < .01; Con: control; PH: prenatal hypoxia

Secreted frizzled‐related proteins (SFRPs) abrogate Wnt signalling by forming complexes with Frizzled family protein to block Wnt signalling. The q‐PCR showed significant Sfrp4 down‐regulation in the foetal forebrain in PH group (*P* < .05), although mRNA expressions of the other 4 Sfrp family members were not changed (Figure 2A). DNA methylation is an important epigenetic mark regulating gene expression. Therefore, 3 Dnmts were examined. In the PH group, a marked increase in mRNA levels of Dnmt1 and Dnmt3b was found (*P* < .05), although Dnmt3a mRNA was not changed (Figure 2B). These results demonstrated that DNA methyltransferases (DNMTs) were up‐regulated in the foetal brain in the PH. Furthermore, 3 regions from CpG islands of Sfrp4 were selected and sequenced (Figure 2C). An increase in the average methylation levels of Sfrp4 was found in the PH group (*P* < .05) (Figure 2D), suggesting that Sfrp4 was hypermethylated in the foetal brain exposed to PH.

### 3.4 Prenatal hypoxia resulted in up‐regulated Wnt/β‐catenin signals in the foetus and down‐regulated in the offspring

**Figure 3:**
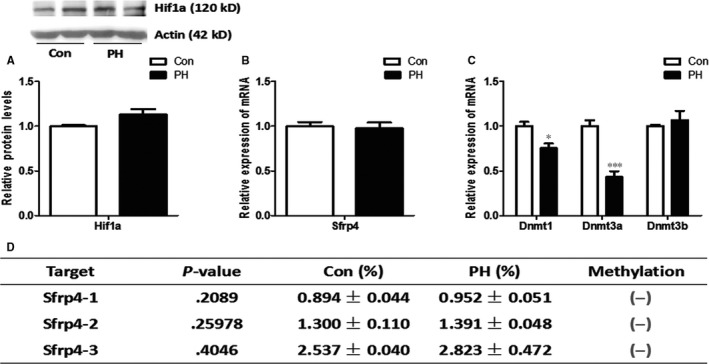
Expression of Hif1a, Sfrp4 and Dnmts in the hippocampus of the adolescent offspring (6 weeks) exposed to PH. A, Hif1a protein showed no difference between the 2 groups. n = 8 from 4 litters. B, Sfrp4 expression was not changed between the 2 groups in adolescent offspring. C, The expressions of Dnmt1 and Dnmt3a were decreased in the hippocampus of adolescent offspring in the PH, whereas Dnmt3b showed no difference. D, The methylation levels of Sfrp4 were unchanged between the 2 groups. n = 10 from 5 litters. *P < .05; ***P < .001; Con: control; PH: prenatal hypoxia

Changes in the genes and proteins between the foetuses and adolescent offspring affected by PH were further compared. Surprisingly, Wnt signalling was changed in the opposite ways between the foetus and offspring (6 weeks). Wnt signalling and Fosl1 that were all increased in the foetal forebrain were decreased in the hippocampus of adolescent offspring (*P* < .05) (Figure S3). Hif1a protein did not change dramatically in the brain of adolescent offspring (Figure 3A). The expression of Dnmt1 and Dnmt3a was decreased in the PH, whereas Dnmt3b did not change (Figure 3C).There were no significant changes in mRNA expression and methylation levels of Sfrp4 gene in the adolescent offspring (Figure 3B, D).

### 3.5 Prenatal hypoxia affected spatial learning and memory in the offspring

In the learning phase, the adolescent offspring rats exposed to PH (6 weeks) showed a significant increase in escape latency from day 3 to day 5 (*P* < .05) (Figure S2A), and the travel distance to the platform was longer compared with the control at day 3 and day 5 (*P* < .05) (Figure S2B). Swim speed was not changed between the 2 groups (Figure S2C). In the probe trail, the number of crossing the platform was less in the PH group compared with the control group (*P* < .05) (Figure S2D). These results confirmed that spatial learning and memory of the adolescent offspring in the PH was damaged.

### 3.6 Up‐regulation of Sfrp4 and down‐regulation of β‐catenin in PC12 cells treated with 5‐aza‐2′‐deoxycytidine

**Figure 4:**
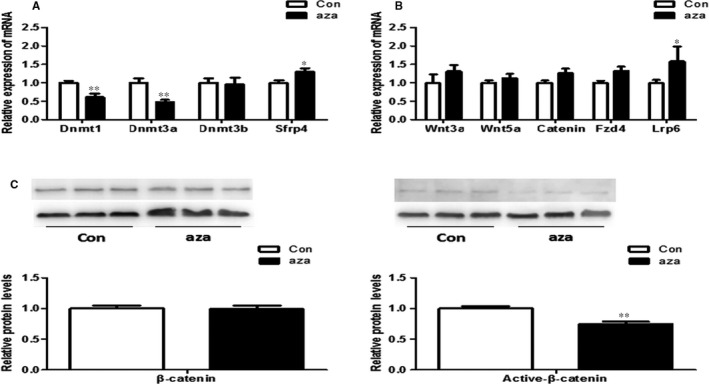
Sfrp4 and Wnt signalling levels in the PC12 cells between the control and the aza group. A, The mRNA expression of Dnmts was decreased, whereas Sfrp4 expression was increased in the aza group. B, mRNA expression of Lrp6 was increased, Wnt3a, Wnt5a, Catenin and Fzd4 was not changed. C, Total β‐catenin was not changed, whereas active‐β‐catenin was significantly decreased in the aza group. n = 6, repeated 2 times. *P < .05; **P < .01; Con: control; aza: PC12 cells treated with aza

The q‐PCR showed that the mRNA expression of Dnmt1 and Dnmt3a was decreased, whereas Sfrp4 was increased in the 5‐aza treated group (*P* < .05) (Figure 4A). Wnt ligands and receptor Fzd4 were unchanged, whereas Lrp6 was increased (*P* < .05) (Figure 4B). The protein level of active‐β‐catenin was down‐regulated, although the mRNA and protein of total Catenin showed no difference between the 2 groups (*P* < .05) (Figure 4C).

## 4 DISCUSSION

This study used a rat model to study the effects of PH on the brain. Birth weight was reduced 20.98% by PH, demonstrating typical IUGR (>10 percentile reduction) following hypoxia. The foetal brain weight was significantly decreased too, showing that PH not only affected general growth in utero, but also delayed brain development. Notably, IUGR led to increased risks of neurodevelopmental impairment during childhood.^27^ In the PH group, the ratio of brain weight to body weight was increased, indicating a relative brain weight in the PH was increased. This could be because of inner mechanisms in protecting important organs in face of threatening risks such as hypoxia. However, despite the ratio showed an protective mechanism might exist, the foetal brain development was still influenced by hypoxia as demonstrated by the reduced weight compared to the control. A couple of immediate and important questions were raised, whether the developing brain could escape from damage by hypoxia except the reduced weight as there was protecting effects? And, if the foetal brain was affected, whether the damaged units in the brain had long‐term influence on later life? In addressing those questions, Wnt signalling was assessed.

Wnt3a and Wnt7a are known to activate Wnt/β‐catenin signalling pathway, and participate in synaptogenesis and brain development.^28^, ^29^ Wnt3a is essential for the early development of hippocampus structures.^30^ Wnt7a plays an important role in regulating excitory synaptic formation.^31^ Wnt7a and Wnt7b are necessary for inducing developmental angiogenesis in the central nervous system.^32^ Wnt/β‐catenin signalling actively participates in the development of prevalent neurological disorders associated with synaptic dysfunction related to Alzheimer's disease, autism and epilepsy.^15^, ^33^, ^34^, ^35^ This study found an up‐regulated Wnt pathway by PH in the foetal forebrain. An increased expression of Wnt receptor, Fzd4, was found, indicating an activated Wnt signalling. It is possible that the activation of Wnt/β‐catenin signalling was as a potential beneficial effect for the foetal brain in face of challenge from hypoxia. Notably, Wnt signalling is regulated by Hif1a. In this study, Hif1a protein was significantly increased in the PH group, strongly suggesting that Hif1a might be involved in the over‐activation of Wnt signalling in the foetal brain during PH.

Secreted frizzled‐related proteins (SFRPs), as negative inhibitors of the Wnt pathway,^36^ play an essential role in cellular proliferation, early development and angiogenesis.^37^, ^38^ Sfrps abrogate Wnt signalling by forming complexes with Frizzled family protein.^39^ Methylation of Sfrps promoter will result in decrease in Sfrps, therefore activate the Wnt pathway.^40^, ^41^, ^42^ Hypoxia was associated with epigenetic changes, including DNA methylation and histone modifications. The formation of m^5^C is catalysed by DNA methyltransferase (Dnmt),^43^ which occurs primarily at cytosine‐guanine dinucleotides (CpGs). Dnmt1, Dnmt3a and Dnmt3b were all expressed during early neurogenesis, playing an important role in neuronal development and maturation.^44^, ^45^ Dnmt3a and Dnmt3b are the de novo DNAMTases that work on non‐methylated DNA, whereas Dnmt1 is the enzyme for the maintenance of methylated DNA.^46^, ^47^ In gene promoters, DNA methylation is related to transcriptional repression.^48^, ^49^ This study found that both Dnmt1 and Dnmt3b were significantly increased in the foetal forebrain in PH group, demonstrating that hypoxia could regulate methylation levels of the genome DNA in the foetal brain. CpG island methylation, along with down‐regulation of Sfrps, has been reported in cancers such as oesophageal carcinoma,^50^ ovarian cancer and mesothelioma.^51^, ^52^ Of 5 Sfrps, Sfrp4 was found with down‐regulated mRNA expression and hyper‐methylation in the foetal forebrain in PH group. To the best of our knowledge, this was the first to demonstrate an altered Sfrp4 demonstrated as epigenetic changes in the forebrain of foetuses following exposure to prenatal insults. In the PC12 cells treated with 5‐aza‐2′‐deoxycytidine, the mRNA expression of Dnmt1 and Dnmt3a was decreased, whereas Sfrp4 was increased, and the Wnt/β‐catenin signal pathway was inactivated. These data suggested that the increased mRNA expression of Sfrp4 might be induced by hypo‐methylation of its promoter and its up‐regulation inhibited the activation of Wnt/β‐catenin signal pathway. These evidence showed that epigenetic silencing of Sfrp4 could be one of the possible mechanisms contributing to the activation of Wnt signalling in the foetal forebrain exposed to prenatal hypoxia. Further study on that issue should be very interesting.

**Figure 5:**
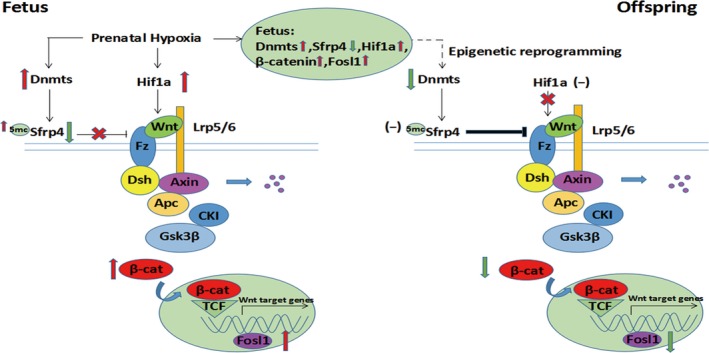
Schematic drawing of epigenetic reprogramming in Dnmts, Sfrp4 and Wnt/β‐catenin pathway from the foetus to offspring

The Wnt pathway is an essential signalling cascade that regulates cellular survival and differentiation, usually working as a positive factor in nervous systems. The Wnt signalling is abnormally activated in the progression of carcinoma, whereas down‐regulated in AD and PD.^15^, ^16^ It is known that PH is one of the most common complications resulting in a redistribution of blood flow which is preferential to the brain. This foetal adaptive response could be considered to protect the brain from hurting and ensure that the brain could keep developing.^53^ Such inner protective mechanisms are quite common in important organs. For example, to avoid excitotoxic cell death, the foetal brain could respond to acute hypoxia/ischaemia by protection mechanisms such as increasing steroid concentrations.^54^ In this study, an increased expression of Wnts was found in the foetal forebrain, providing new evidence that molecular changes might be involved in protective processing for the organ in face of hypoxia insult. The most interesting part of this finding is that: despite existence of protective processing in the brain against hypoxia during pregnancy, the expression of Wnt signalling in the hippocampus was suppressed in the adolescent offspring, which was opposite to that found in the foetal forebrain. The suppressed Wnt signalling associated with abnormal behaviours in this study were consistent to that reported before.^26^ A reduced activity of central Wnt pathway has been shown in affecting behavioural changes.^55^ Clinically, such kinds of behavioural changes in the adolescent rats might be able to be translated into the link for behavioural problems in children such as hyperactivity. Why prenatal hypoxia‐produced increase in Wnt activities in the foetal forebrain could be turned into the opposite decreased levels in the offspring brain is a wonderful scientific question in further exploring secrets for harmful factor‐induced brain damage in developmental origins. One explanation is that over‐activity of molecular responses to PH in developing brain might be a double‐edged sword, not only as protective processing, but also leading to damage of nervous cells as well as molecules inside and outside cells. In this study, Sfrp4 was unchanged in the hippocampus between the control and PH offspring (6 weeks). This negative finding is as important as the positive data of the decreased Sfrp4 in the foetal forebrain. As the down‐regulated Sfrp4 associated with up‐regulated Wnts, Wnt/β‐catenin and Fosl1, was considered as epigenetic gene which was involved in protective processing in the foetal brain, the unchanged Sfrp4 linked to decreased Wnt3a, Wnt/β‐catenin and Fosl1 in the offspring hippocampus could serve as a new evidence from opposite angle (Figure 5), showing epigenetic reprogramming could be participated in the regulation of both protective and harmful processing in the foetal and offspring brain. It is impossible to get the foetal hippocampus for protein analysis from rats as the size is too small. Thus, using foetal forebrain that contains hippocampus in the comparisons.

In summary, prenatal hypoxia significantly affected foetal brain development and caused activation of the Wnt signalling, which might be partly mediated by an increased Hif1a and functional loss of Sfrp4 through DNA methylation. This is the first time to demonstrate that Sfrp4‐mediated epigenetic methylation may be involved in possible protective mechanisms by the activation of Wnt/β‐catenin against hypoxia in the foetal brain. Notably, disadvantageous effects of the over‐activity of Wnt/β‐catenin signalling were also linked to a negative change in Sfrp4 associated with an suppressed Wnt/β‐catenin signalling later in the offspring brain. The new information gained contribute to further understanding the mechanisms underlying development of certain brain diseases and benefiting further investigation of early prevention of those health problems.

## CONFLICT OF INTEREST

The authors confirm that there are no conflicts of interest.

## AUTHOR CONTRIBUTION

Yingying Zhang, Mengshu Zhang, Bin Wei, Axin He and Lingjun Li, Likui Lu performed the research, Yingying Zhang, Mengshu Zhang, Xiang Li and Miao Sun analysed the data and wrote the paper, Miao Sun and Zhice Xu contributed essential reagents or tools. Miao Sun, Zhice Xu and Lubo Zhang designed the research study and wrote the paper.
