# Supplementary material for: Methylation‐reprogrammed Wnt/β‐catenin signalling mediated prenatal hypoxia‐induced brain injury in foetal and offspring rats
Source: J Cell Mol Med. 2018 May 28;22(8):3866–74. doi: 10.1111/jcmm.13660 (PMC6050486; doi:10.1111/jcmm.13660)
Supplement: Supplementary file 1 [file JCMM-22-3866-s001.docx]

**Supplemental Data**

**Methylation-reprogrammed Wnt/β-catenin signaling mediated prenatal hypoxia-induced brain injury in fetal and offspring rats**

Yingying Zhang^1,#^, Mengshu Zhang^1,#^, Lingjun Li^1,#^, Bin Wei, Axin He, Likui Lu, Xiang Li^1^, Lubo Zhang^1,2^, Zhice Xu^1,2,^*, and Miao Sun^1,^*.

**Table S1. Primers used in this study**

| **Primer Name** | **Primer Sequence** |
| --- | --- |
| Wnt2-F | CAA CAT TGA CTA CGG GAT CAA A |
| Wnt2-R | CCA TGA CAC TTG CAT TCT TGT T |
| Wnt3a-F | AAC CGT CAC AAC AAT GAG GC |
| Wnt3a-R | GCA GGT CTT CAC TTC GCA AC |
| Wnt5a-F | AAG CAG GTC GCA GGA CAG TA |
| Wnt5a-R | GAG TTG AAG CGG CTG TTG AC |
| Wnt7a-F | CCG AGA GAT CAA GCA GAA TGC |
| Wnt7a-R | TCC AGT TTC ATG TTC TCC TCC A |
| Wnt7b-F | CAA GAG CTC CGA GTA GGG AGT C |
| Wnt7b-R | ACA GCC ACA GTT GCT CAG ATT G |
| Catenin-F | GCT TTG CTC AAC AAA ACA AAC G |
| Catenin-R | ACA GAC AGC ACC TTC AGC ACT C |
| Fzd4-F | GCT ACA ACG TGA CCA AGA TGC |
| Fzd4-R | ATG GGG ATG TTG ATC TTC TCT G |
| Lrp6-F | GTT GTA AGC TCG GTT CCA AAT C |
| Lrp6-R | CAA TTT TGG GAG ATC TTT CCT G |
| Fosl1-F | TAA GTG CAG AAA CCG AAG AAA GG |
| Fosl1-R | TGT CCT TCT TGT CTT CTT CTG GG |
| Sfrp1-F | CTG AGG TTG TCC AGC TGG TG |
| Sfrp1-R | GAC AAC GAG TTG AAA TCG GAG G |
| Sfrp2-F | CCA AGG TGT GTG AAG CCT GC |
| Sfrp2-R | AGC ACT GAT TTC TTC AGG TCC CTA TC |
| Sfrp3-F | TGA TTC TGA GTG GAA TCG CTG |
| Sfrp3-R | CCT CAG TGT TAA TGA GGA GTA TGT CAT C |
| Sfrp4-F | GAC CGT GGA GTT TGT ATC TCT CCA G |
| Sfrp4-R | TTG CTC AGG TAT GTT GCC AGG |
| Sfrp5-F | GTT CTT CAT GTG CAG AAC CAG C |
| Sfrp5-R | TGA GAT CGA GCA CAG TGC TGA C |
| Dnmt1-F | GCT CCA GTA GAG CAG GTT GAT GTC |
| Dnmt1-R | ATC AAA GGA AGC AAC CTG GAC G |
| Dnmt3a-F | TCA TGA CAG CGA TGA AAG TGA C |
| Dnmt3a-R | CCA CAT GTC GGT GTA AAC TTC C |
| Dnmt3b-F | TCC TGG AAA GCC ACC TCC AAG |
| Dnmt3b-R | GCT GGC TGA ACA GAC CCA GAG |
| Actin-F | CCT AAG GCC AAC CGT GAA AAG |
| Actin-R | GCT CGA AGT CTA GGG CAA CAT AG |

**Table S2. Blood oxygen level and oxyhemoglobin saturation in fetal blood**

|  | Con | PH |
| --- | --- | --- |
| PH | 7.25±0.01 | 7.23±0.01 |
| PO_2_(mmHg) | 53.67±3.74 | 42.37±3.48* |
| PCO_2_(mmHg) | 54.13±2.63 | 55.09±3.12 |
| SO_2_(%) | 58.89±1.87 | 47.64±2.17* |

n=10; *, p<0.05; Con: control ; PH: prenatal hypoxia.

**Figure S1. Fetal brain and body weight**

Fetal brain and body weight were measured at GD 21. Prenatal hypoxia reduced brain and body weight, indicating growth delay and poor brain development. The ratio of brain weight to body weight in the PH was increased. n=63 from 5 litters; *, p<0.05; ***, p<0.001; Con: control ; PH: prenatal hypoxia.


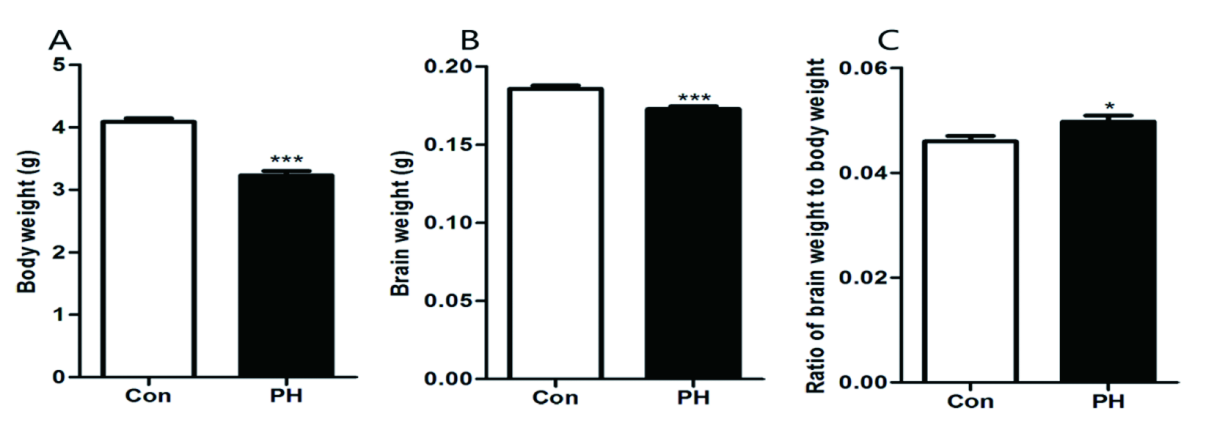


**Figure S2. Spatial learning and memory in the offspring between control and PH group**

Morris water task showed prenatal hypoxia damaged the spatial learning and memory in the offspring (6 weeks). The PH offspring showed a longer escape latency and travel distance from the 3th to 7th day. No difference in the swim speed between the two groups. Time of crossing the target areas were decreased in the PH group. n=8 from 4 litters; *, p<0.05; **, p<0.01; ***, p<0.001; Con: control; PH: prenatal hypoxia.


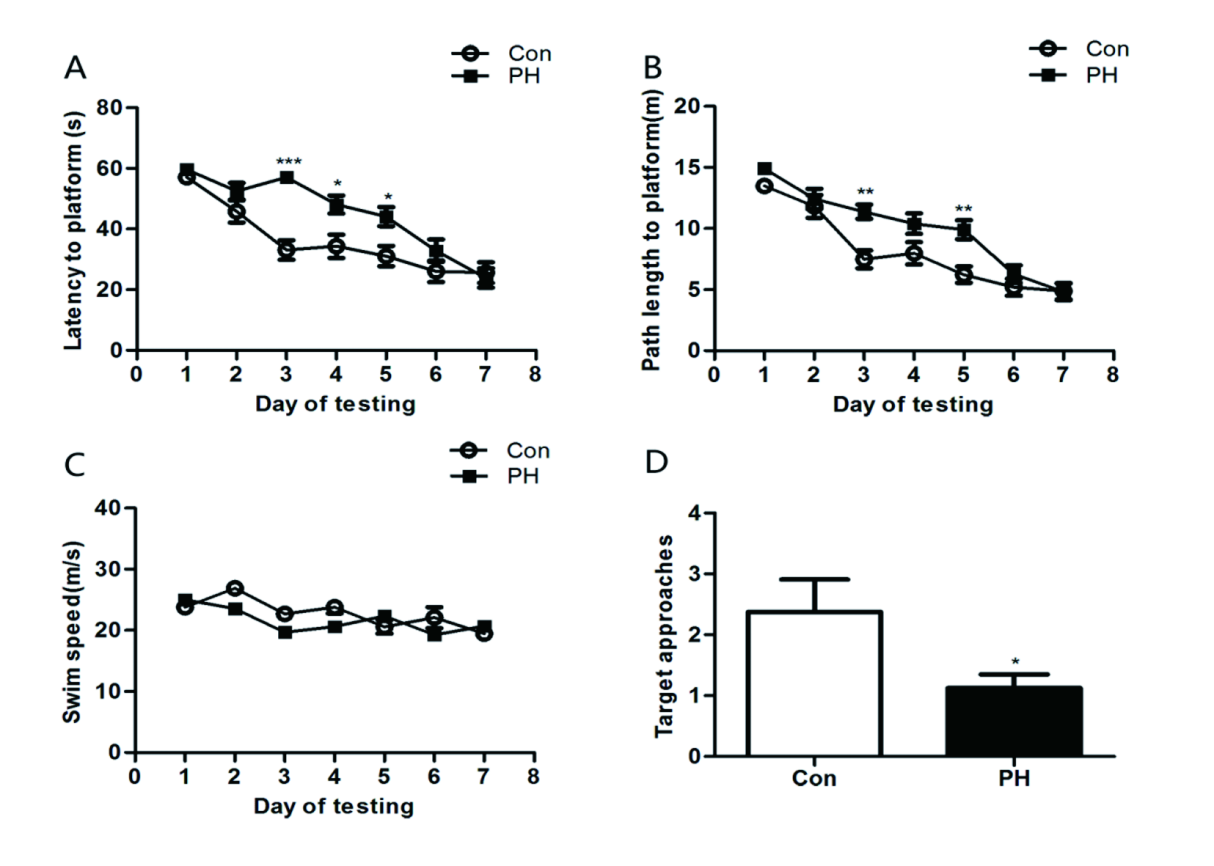


**Figure S3. Expressions of Wnt3a, Catenin, Fzd4, Lrp6 and Fosl1 in the hippocampus of adolescent offspring**

The q-PCR showed that Wnt signaling was down-regulated in the brain of adolescent offspring (6 weeks) in the PH group. n=10 from 5 litters; *, p<0.05; Con: control; PH: prenatal hypoxia.

**
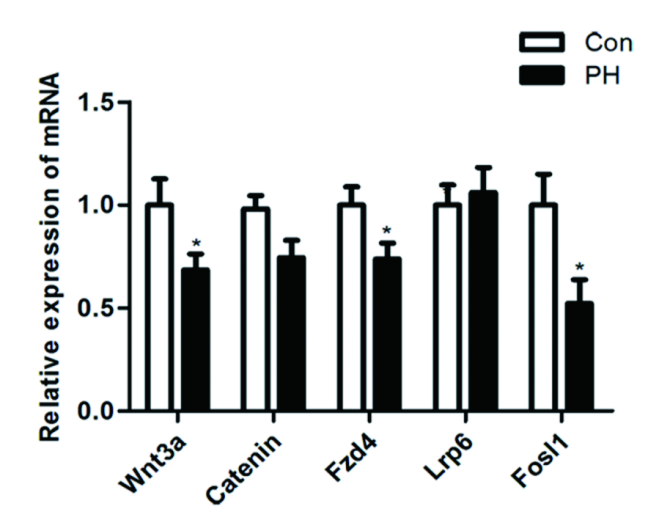
**
